# Preventive Measures and Crowd Management Strategies to Mitigate the Risk of Stampedes in Public Gatherings: A Systematic Review

**DOI:** 10.3389/phrs.2026.1609310

**Published:** 2026-06-18

**Authors:** Rahul Singh Chowhan, Ramesh Kumar Hudda, Arun Kumar Sharma, Pankaj Bhardwaj

**Affiliations:** 1 ICMR - National Institute for Implementation Research on Non-Communicable Diseases, Jodhpur, India; 2 Department of Community Medicine, UCMS, New Delhi, India

**Keywords:** crowd management, emergency evacuation, human stampedes, public safety, risk mitigation in crowds

## Abstract

**Objectives:**

To evaluate crowd-management strategies used at mass public gatherings and to identify the evidence gaps and priorities that reduce the risk of stampedes.

**Methods:**

Following PRISMA 2020 and PRISMA-S guidelines, the paper searched PubMed, Scopus, Web of Science, Google Scholar (gray literature), and the WHO Global Health Library (gray literature) published between January 1, 2010 and December 30, 2025. Eligible studies included observational, quasi-experimental, and simulation-based evaluations of crowd-safety interventions. Two reviewers independently screened records, extracted data, and appraised study quality using ROBINS-I and JBI checklist. A narrative (thematic) synthesis grouped interventions into engineering, operational, technology, and communication approaches.

**Results:**

Of 833 unique records identified, 13 studies met the inclusion criteria. Included studies were mainly observational analyses, simulation/modeling studies, or quasi-experiments from diverse settings (pilgrimages, festivals, sports events). Multi-modal strategies combining environmental design (one-way flows, barriers), operational controls, real-time monitoring, and trained personnel were consistently associated with improved crowd-flow indicators (shorter evacuation times).

**Conclusion:**

Integrated crowd-management approaches can mitigate stampede risks, but require high-quality empirical evaluations and standardized outcome metrics to strengthen evidence for policy and practice.

**Systematic Review Registration:**

https://www.crd.york.ac.uk/PROSPERO/view/CRD420251142906, identifier CRD420251142906.

## Introduction

Crowd disasters/stampedes remain a recurrent global threat, particularly during large public gatherings such as religious pilgrimages, festivals, concerts, political rallies, and sporting events. Incidents of crowd-related disasters often stem from factors such as panic, severe congestion, and poor crowd management, leading to significant injuries and fatalities [1]. There are notable tragedies, including the 2015 Hajj stampede in Saudi Arabia and the 2021 Astroworld Festival crowd surge [2], that highlight the urgent need for effective preventive measures at large events. From the standpoint of public health, stampedes pose serious concerns, as they not only result in immediate physical injuries but also contribute to long-lasting psychological effects, strain emergency response systems, and disrupt local healthcare services. Vulnerable populations, including children, older persons, and individuals with disabilities, face heightened risks during such emergencies due to their lack of preparedness. Consequently, it has become increasingly vital to incorporate crowd safety into urban planning, public health strategies, and emergency response frameworks, especially amid rapid urbanization and the rise of mass gatherings worldwide [3].

Over the past 20 years, various crowd management techniques have been developed to improve safety at public events. These strategies encompass the use of physical barriers, the creation of specific entry and exit points, the deployment of trained security personnel, and the establishment of real-time monitoring systems [4]. Additionally, organizations such as the World Health Organization and the United Nations have emphasized the importance of thorough pre-event planning and systematic risk assessments to prevent crowd-related incidents [5]. Yet, despite these improvements, stampedes still occur, raising concerns that current strategies are either not effective enough, inconsistently implemented, or inadequately evaluated in real-world settings. The existing research landscape lacks cohesion, as most studies focus on descriptive case studies. Additionally, these studies lack robust assessments to determine how effectively different strategies work together. Furthermore, the lack of a broader perspective and limited comparisons across event types and regions has created significant gaps in our global understanding of best practices [6]. This fragmented approach prevents the creation of unified, evidence-based, and ubiquitous guidelines, slowing the development of consistent, easy-to-implement crowd safety measures.

Previous reviews on crowd disasters have primarily focused on descriptive analyses of individual incidents or on specific event types such as sporting events or religious gatherings. However, these reviews have rarely synthesized empirical, observational, and simulation-based evidence together to evaluate the effectiveness of preventive strategies across diverse mass-gathering contexts.

The systematic review directly addresses this research gap by examining the preventive measures and crowd-control methods used to prevent stampedes at large public gatherings. The main objective of this review is to assess the quality of existing research and identify preventive strategies that are practical, effective, and feasible in a real-world setting. The research question is, “Which preventive measures and crowd management strategies are functional and efficient in mitigating both the recurrence and seriousness of stampedes at large public gatherings?”

## Methods

### Review Protocol and Registration

This review was conducted in accordance with the Preferred Reporting Items for Systematic Reviews and Meta-Analyses guidelines [7]. This systematic review adhered to a predefined registered protocol in the International Prospective Register of Systematic Reviews (PROSPERO) under registration number CRD420251142906. The methodology followed the PRISMA 2020 and PRISMA-S guidelines for transparent reporting of systematic reviews and search strategies.

### Search Strategy

A comprehensive search using PubMed, Scopus, Web of Science, Google Scholar, and the World Health Organization (WHO) Global Health Library databases was conducted to identify studies between 01 January 2010 and 30 December 2025. The literature search aimed to identify articles that reviewed crowd management, mitigation, and safety plans for stampedes and mass gatherings. Only studies published in English were included due to feasibility constraints in screening and data extraction. The usage of relevant keywords and controlled vocabulary was considered in all the databases as follows: (“stampede” OR “crush” OR “crowd” OR “mass gathering” OR “large events”) AND (“safety” OR “crowd management” OR “risk mitigation” OR “emergency preparedness” OR “public safety”). The detailed search strategy was based on the PRISMA-2020, for which the checklist is provided in Supplementary Material S1, and the search strategy is provided in Supplementary Material S2, with the databases searched, date limits, and the number of records retrieved in each database. Reviews, editorials, opinion pieces, and conference abstracts without full empirical data were excluded.

### Inclusion and Exclusion Criteria

The PICOS framework was used to determine the scope and relevance of studies conducted to follow eligibility and selection procedure regulations, and to define the scope of this review. The Population (P) consisted of individuals attending mass public gatherings, such as religious pilgrimages, music festivals, sporting events, and political rally settings, where the risk of crowd crushes or stampedes is high [8]. The Intervention (I) focused on preventive or crowd-management strategies designed to reduce the likelihood or severity of stampedes. These included physical design modifications (e.g., one-way flow systems, barrier placements), operational strategies (e.g., crowd density control, entry/exit flow management), technological tools (e.g., real-time surveillance, AI-based crowd simulations), and communication-based interventions (e.g., public announcements, signage, and emergency drills). The Comparator (C) included standard practices in crowd management, or, where applicable, specific contexts that lacked formal intervention. The Outcomes (O) included both direct and proxy measures of safety effectiveness, such as reduced incidence of crowd crushes or stampedes, improved crowd flow, increased evacuation efficiency, and reduced density-related congestion [9]. Lastly, the Study design (S) encompassed a broad range of empirical evidence, including observational studies, quasi-experimental designs, simulation-based modeling studies, and retrospective analyses of real-world events. Simulation and modeling studies were included to gain insights into crowd dynamics and testing of preventive strategies under controlled and high-risk conditions that are not feasible in real-world experimental settings. Such an extensive set of PICOS structures ensured the inclusion of a wide range of applicable evidence in the synthesis of best practices for stampede prevention and crowd safety.

### Study Selection

**FIGURE 1:**
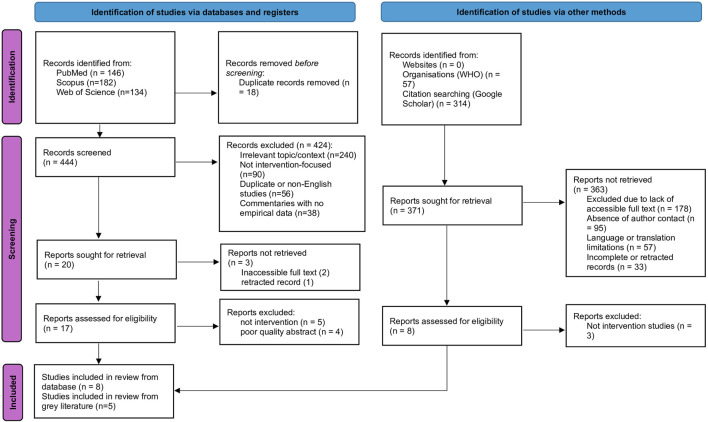
PRISMA 2020 flow diagram of study selection. Stampede Prevention Review, Global, 2010–2025.

Following the literature search, all retrieved records were managed systematically to ensure transparency and reproducibility. The screening was conducted using a multi-phase approach: Phase 1 involved the removal of duplicate records across databases; Phase 2 involved screening of titles and abstracts against eligibility criteria and predefined keywords (e.g., “stampede,” “crowd management,” “risk mitigation”); Phase 3 involved further screening of the contents of articles by reading the full text using the PICOS framework; Phase 4 involved the assessment of outcome levels to select those that reported primary or secondary outcomes related to crowd safety (e.g., evacuation time, crowd density); and Phase 5 involved the inclusion of relevant gray literature (e.g., WHO Global Health Library) that reported applicable prevention strategies. The screening process was conducted independently by two reviewers (RSC and AS) to identify studies that met the inclusion criteria. A third reviewer was consulted to resolve any discrepancies or disagreements between the two reviewers. Reasons for exclusion (e.g., wrong context, no evaluation of prevention strategy) were recorded at the full-text stage. The study selection process adhered to the Preferred Reporting Items for Systematic Reviews and Meta-Analyses (PRISMA 2020) guidelines. The study selection process is summarized in the PRISMA flow diagram (see Figure 1).

### Risk of Bias in Non-Randomized Studies of Interventions

In this review, the methodological quality of the articles was evaluated using the Cochrane Risk Of Bias In Non-randomized Studies-of Interventions (ROBINS-I) [10]. The considered biases included random participant assignment, departures from intended interventions, missing outcome data, outcome measurement bias, bias in the selection of reported results, and the risk of bias in general.

### Handling of AI and ML in Simulation-Based Crowd Management

Emerging applications of artificial intelligence (AI) and machine learning (ML) have shown potential in enhancing crowd monitoring and prediction capabilities. Evidence from recent studies suggests that AI-based surveillance and predictive modeling can help identify high-density zones and improve crowd-flow management. For example, in a simulated case study, a convolutional neural network (CNN)-based crowd surveillance system accurately detected areas of critical density in real time with 93% accuracy [11]. Another modeling-based study has applied a predictive ML model trained on historical stampede data from Mecca and India to optimize gate scheduling during entry/exit events, resulting in a 27% reduction in simulated congestion time [12].

The scope of AI has recently expanded beyond physical sensors to include the sentiment analysis of social media data, offering a transformative tool for predicting crowd behavior by detecting shifts in the “emotional state” of the gathering. Furthermore, recent field evaluations have transitioned these technologies from simulation to large-scale reality; the 2025 Maha Kumbh Mela successfully utilized AI-powered density surveillance and drone-assisted evaluations, integrated into a centralized command center, to manage record-breaking crowd densities [13, 14].

While these findings highlight the potential of AI/ML to improve anticipatory and adaptive crowd safety strategies, they were interpreted as predictive or exploratory evidence rather than directly generalizable real-world outcomes. During thematic synthesis, AI/ML-based studies were primarily mapped to the theme of crowd monitoring and technology, while also contributing insights into predictive and adaptive crowd management strategies.

### Data Extraction

From each included study, we extracted details on authors, year, country, setting/event type, study design, sample size, description of the intervention(s) or strategy, comparator (if any), outcomes measured (e.g., crowd density metrics, casualty reduction, evacuation time), and key results are provided in Supplementary Material S3. One of the reviewers extracted the data using a standardized extraction form, which was verified by the other. Given the diversity of included studies, outcome measures were heterogeneous. They included both direct indicators (e.g., incidence of crowd crush or injury) and proxy indicators (e.g., crowd density, evacuation time, congestion levels, and situational awareness). These outcomes were synthesized narratively to allow comparison across varied study designs.

### Quality Assessment

Quality appraisal was conducted using two design-specific tools. The ROBINS-I tool was applied to seven non-randomized intervention and simulation studies, generating domain-level ratings from Low to Critical risk of bias [13, 15–20] (see Supplementary Material S5). The JBI Critical Appraisal Checklist for Analytical Cross-sectional Studies was applied to six observational and descriptive studies, producing criterion-based Yes/No/Unclear judgments [14, 21–25] (see Supplementary Material S6). Results from both tools were synthesized qualitatively to inform study weighting in the narrative synthesis (see Supplementary Material S7).

### Data Synthesis

A quantitative meta-analysis could not be conducted because the included studies were highly heterogeneous in design, context, methodology, and outcome metrics. Hence, a thematic (narrative) synthesis method was used. The interventions were grouped into engineering controls, operational interventions, technology-enabled solutions, and communications/awareness measures. Each theme followed a deductive coding approach in which intervention categories were predefined based on commonly reported domains in the crowd-management literature. Themes were interpreted to determine the direction and strength of the reported effects, allowing commonalities across interventions to be identified. When available, relevant quantitative information (e.g., mean differences, simulated density reductions, evacuation times, or effect sizes) was summarized to strengthen and accompany the qualitative synthesis. Two reviewers, using a deductive approach based on predefined, theory-driven themes identified from the literature, independently performed thematic coding of the included studies. Data extraction and quality assessment were also conducted independently to ensure methodological rigor. Discrepancies at any stage were discussed and resolved through consensus; where disagreements persisted, a third reviewer adjudicated. to ensure consistency in classification.

A study-weighting framework was applied to reflect each study’s relative contribution to the overall synthesis. Each study’s weight was derived from (1) methodological quality based on JBI or ROBINS-I appraisal, (2) data richness (empirical or high-fidelity simulation evidence), and (3) relevance to the review objectives. Scores were assigned on a 1-3 scale for each factor, normalized to a total weight of 1.00 across all included studies. These weights were not used for statistical pooling but to visually indicate each study’s influence within the narrative synthesis (see Figure 5).

## Results

### Study Selection

In total, 833 articles were identified through database and gray literature searches, comprising 146 from PubMed, 182 from Scopus, 134 from Web of Science, and 371 from gray literature sources, including the WHO (57) and Google Scholar (314). After removing 18 duplicates, 815 records remained for screening based on titles and abstracts. During this phase, 424 records were excluded because 240 had an irrelevant topic or context, 90 were not intervention-focused, 56 were duplicates or non-English studies, and 38 were commentaries or editorials lacking empirical data. Following this screening, 391 reports were sought for retrieval, but 366 were not retrieved due to a lack of accessible full text (178), a lack of author contact response (95), language or translation limitations (60), or incomplete or retracted records (33). Consequently, 25 reports were successfully retrieved and assessed for eligibility. Twelve citations were excluded at the full-text stage: eight failed to address stampede prevention strategies specifically, and four were excluded due to low methodological quality. Ultimately, 13 studies met all inclusion criteria and were included in the systematic synthesis (see Figure 1).

### Characteristics of Included Studies

The essential characteristics of the 13 studies included in this review are presented in Supplementary Material S4. The publication years range from 2010 to 2025, and recent years show more publications. Geographically, studies originated mainly from Asia and the Middle East (n = 10; India, China, South Korea, Egypt, Saudi Arabia, and Iran), with two from the United States and one from the United Kingdom. Event settings included religious pilgrimages (e.g., Hajj, religious festivals), sports and music events, urban festivals, and transportation hubs. Study designs were heterogeneous: three simulation or modeling studies, two experimental or quasi-experimental evaluations, three retrospective observational analyses or reviews, and five descriptive case studies or field evaluations that used mixed-methods and contextual analysis. Most of the results presented in the articles considered composite outcomes, including measures of crowd density, evacuation time, or near-miss incidents; specific injury rates or stampede occurrences were reported in a minority of the studies. Common interventions included one-way pedestrian flows, physical barriers/fencing, crowd capacity limits, increased security staffing, crowd-monitoring systems (CCTV and mobile data), emergency preparedness drills, and emergency communication measures (signage and audio announcements). Randomized controlled trials were not identified; evidence was primarily derived from observational or modeling research.

### Methodological Quality

Methodological quality appraisal results are presented in Supplementary Material S5 (ROBINS-I), which summarizes key study characteristics, and in Supplementary Material S6, which presents JBI checklist assessments. Overall, three studies were judged high quality, four moderate, and six low methodological quality. The most frequent limitations included the absence of control or comparator groups (particularly in observational and descriptive designs), small or non-random participant samples, and potential confounding factors such as environmental or organizational influences that were not statistically controlled.

Many studies did not use clearly defined data-collection procedures or validated measurement tools. Simulation-based studies demonstrated relatively greater methodological rigor, as their models were calibrated against real data [15, 18]. In contrast, descriptive and case-based studies [21, 22] provided contextual insights but were hampered by their non-experimental nature and limited methodological detail. Empirical field evaluations conducted under real conditions [17, 23] provided important evidence from a practical perspective, although they varied in terms of analytical transparency and external validity.

No randomized controlled trials (RCTs) were found; therefore, the focus of the methodological evaluation was on the appropriateness of the design, the reliability of the data, and the clarity of reporting, rather than on experimental comparisons. Detailed quality appraisal findings are available in Supplementary Materials S5, S6, and the ROBINS-I–based bias and narrative-synthesis results as illustrated. (see Figures 3, 4).

### Themes in Preventive Strategies

Through the studies, the analysis identified several thematic categories of preventive measures. Key findings are summarized below, with illustrative examples. An evidence-mapping matrix was also developed to visualize the distribution of study designs, intervention strategies, and reported outcomes across the included studies, as described in Supplementary Material S7. This provides a structured visualization of preventive interventions for crowd safety and management. The map organizes studies along two primary dimensions: intervention (rows), such as “Evacuation Algorithms and Simulation,” “Spatial Planning,” and “Inter-organizational Communication,” and outcomes (columns), like “Crowd Safety,” “Evacuation Efficiency,” and “Situational Awareness.” Each colored circle represents one or more studies that have investigated a specific intervention-outcome pair, with the color indicating the study design: green for Simulation (SIM), light blue for Experimental (EXP), dark blue for Observational (OBS), and purple for Review/Practice Survey (REV). It also reflects the confidence level of supporting evidence (see Supplementary Material S8).

#### Theme 1: Built Environment and Spatial Risk Design

There are studies that have focused on engineering and infrastructure control design solutions. The general strategies were single-directional flow systems, controlled entry/exit facilities, and the installation of barriers. Simulation-based studies have demonstrated that implementing one-way corridors or controlled entry systems, such as turnstiles, can effectively manage crowd flow as a strategy to alleviate local congestion. For example, the study highlighted that proper management of crowd density by eliminating bottlenecks and providing adequate space, along with the use of path barriers and widened walkways, is crucial. Major changes include improving infrastructure at pilgrimage sites, expanding bridges, and adding multiple exits, which have reduced the risk of stampedes [26]. Recent evidence distinguishes spatial risks by organizational type, noting that while organized events see higher risks on stairs, spontaneous gatherings face significantly greater danger at exit points [24]. Furthermore, a retrospective analysis of mass gatherings suggests that a series of structural failures, rather than irrational crowd panic, are the primary underlying causes of disasters at religious sites [25].

#### Theme 2: Operational Management, Planning, and On-Ground Measures

There are studies that also prominently featured organizational stampede prevention strategies. Keeping overall capacity limits and allowing certain entry times helped evenly distribute crowd inflow throughout the events. For example, in one study [21], it was found that staggered start times during a marathon effectively reduced the sudden surge in the crowd. Additionally, a contingent plan, like the development of evacuation protocols and regular emergency drills, was noted in several studies. Another study found that preparedness exercises have significantly improved coordination and communication among security personnel, enabling a reflexive response during simulated emergencies [17]. Interagency collaboration among police, fire, and event management teams was consistently emphasized, advocating for a “whole-of-society” approach that seeks participation of all stakeholders. Collectively, proactive and pre-event planning emerged as a recurring need and an essential determinant of crowd safety, rather than reactive, *ad hoc* responses. The success of operational strategies is closely linked to behavioral predictability and adherence to instructions. In highly structured events, such as marathons or ticketed gatherings, controlled entry systems are more effective. In contrast, spontaneous or emotionally driven gatherings may exhibit irregular inflow patterns, limiting the effectiveness of pre-planned scheduling strategies [27]. Modern frameworks now advocate a formalized five-stage risk reduction paradigm spanning from initial decision-making and inter-agency approval to final integration, to replace inefficient *ad hoc* responses [25]. The implementation of a highly structured seven-tier security architecture during the 2025 Maha Kumbh Mela successfully managed record-breaking densities of over 660 million devotees, demonstrating the scalability of tiered operational planning [13].

#### Theme 3: Real-Time Surveillance and Monitoring Systems

Some studies have highlighted the growing importance of real-time monitoring in crowd management. A Unity-based simulation platform has been developed with a visualization tool for viewing crowd density and for identifying potential risk zones within the monitored area [18]. This simulation system allows event planners to test and evaluate “what-if” conditions (e.g., adding crowd barriers, route modifiers, and signboards) to assess their impact on crowd flow. Comparable technologies, such as CCTV and cellular data, can be used side-by-side to understand the situation. For example, one study used mobile network signals to monitor crowd gatherings in Itaewon before the crush, underscoring the importance of prior notification as an early warning system [28]. Hence, by combining technological systems, predictive insights can be obtained alongside getting operational flexibility to prevent overcrowding. However, the effectiveness of physical design interventions is highly dependent on user compliance and crowd behavior. In situations where individuals deviate from designated pathways or attempt to override barriers during panic conditions, the intended benefits of engineered flow control may be reduced. Additionally, integrating underwater sonar, drone-assisted evaluations, and AI-powered density surveillance into a centralized Integrated Command Center (ICC) has proven effective in providing the situational awareness necessary for rapid intervention in high-density scenarios [13].

#### Theme 4: Workforce, Training and Human Factors

Other studies have shown that human resource management is as important as technical crowd control measures. Trained attendants, police personnel, and volunteers have been instrumental in maintaining order and facilitating the flow of people. Where personnel were deployed at potential bottlenecks, such as narrow passageways or stairs, crowd movement remained relatively balanced, and panic incidents were reduced. One study also found that events where trained marshals were deployed had significantly fewer incidents of sudden scuffles [29]. Another study found that inadequate training often leads personnel to react late or lose coordination in stressful situations. Consequently, it has been suggested that a uniform training program for all field personnel, covering topics such as crowd psychology, first aid, risk identification, and emergency response, is necessary so that all teams can operate with a uniform understanding and preparation at large events [28]. Evidence suggests that stationing trained attendants and security at high-risk bottlenecks, such as stairs and narrow passageways, is instrumental in balancing crowd movement and reducing scuffles. However, the efficacy of these personnel depends on standardized training in crowd psychology and risk identification, as inadequate preparation often leads to coordination failures under stress. Psychological insights further reveal a “follower effect,” where individuals are more likely to follow surrounding people than physical signage, while recent analysis of global overcrowding incidents identifies specific risk factors where individual behaviors like tripping or pushing can rapidly escalate localized congestion into a lethal stampede [24].

#### Theme 5: Inter-Agency Coordination and Community Education

Effective public communication has repeatedly been highlighted as a key element in crowd management. Several studies have shown that simple measures—such as clearly indicating entry and exit routes and making periodic announcements via loudspeakers—helped guide people and prevent confusion [19, 21]. Two studies tested early warning or “crowd alert” systems, which provided information about potential delays or danger via SMS messages. Both studies found that such information was effective in evenly dispersing crowds and reducing pressure in congested areas. These studies specifically suggested that issuing real-time public warnings about crowd density in confined or narrow spaces and directing pedestrian flow could significantly reduce the likelihood of stampedes [28, 29]. Social media platforms are increasingly recognized as vital channels for issuing real-time public warnings and 'crowd alerts' to help disperse congested areas. However, these systems require robust mitigation strategies to prevent the spread of misinformation during emergencies [14].

#### Theme 6: Governance, Policy and Regulatory Measures

**FIGURE 2:**
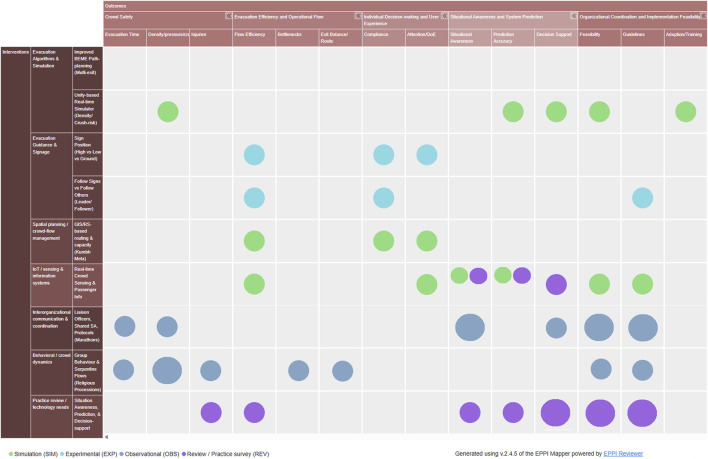
Evidence Gap Map of intervention-outcome relationships in crowd management. Global, 2010–2025.

Only a few studies have analyzed the broader policy aspects of crowd control and management, focusing on legal provisions and regulatory controls. Another study from the Middle East has noted that enforcing more stringent event permit requirements and refraining from any unauthorized crowd gatherings can help in curbing the uncontrollable situation. Although evidence on this topic is limited, many experts agree that crowd safety protocols should be prepared, checked, verified, and enforced at the public gathering site, and that financial investment in facilities and infrastructure should be substantially increased [26, 29]. The study also pointed out that event organizers often spend too little on security planning resources and also put forth the need for dedicated funding for preventive measures and research, including the use of modern technologies such as an artificial intelligence-based real-time crowd monitoring system [27]. (Insert Figure 2 here).

### Risk of Bias Assessment

**FIGURE 3:**
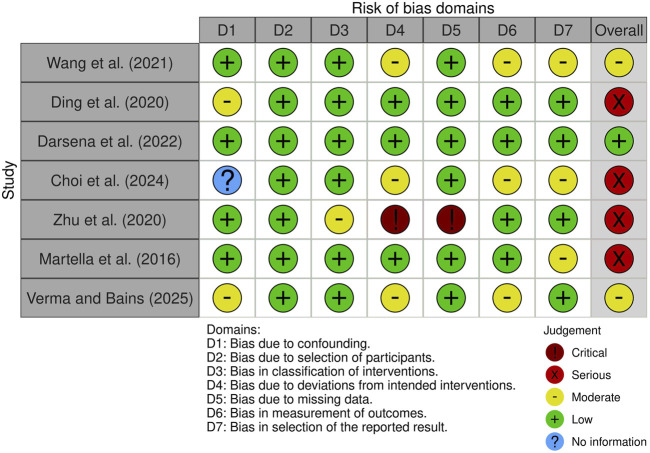
Visual risk of bias assessment of included studies using ROBINS-I. Global stampede intervention studies, 2010–2025.

**FIGURE 4:**
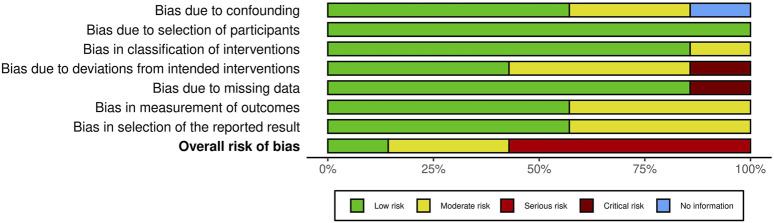
Overview of bias-risk distribution across studies. Global, 2010–2025.

The Risk of Bias In Non-randomized Studies-of Interventions (ROBINS-I) evaluation of seven studies revealed a range of methodological quality. Only one study (Darsena et al., [17]) was judged to have a low overall risk of bias due to its robust design and clear outcome measurement. Two studies (Wang et al. [15] and Verma and Bains [13]) were classified as moderate risk, primarily due to concerns regarding confounding and deviations from intended interventions in field or simulation settings. The remaining four studies (Ding et al.; Choi et al.; Zhu et al.; and Martella et al., [15, 18, 19, 20]) were found to have a serious risk of bias, often stemming from issues in intervention classification and outcome measurement. Simulation-based research frequently exhibited limitations in generalizability, while experimental and field designs faced challenges with reporting bias and deviations from standard protocols. The results are presented as traffic light plots and summary plots, as shown in Figures 3, 4, respectively, for critical appraisal of the studies (Insert Figures 3, 4 here).

### Results of Narrative Synthesis

The included evidence from 13 studies demonstrates that multi-modal crowd control frameworks combining structural, technological, and procedural strategies play a vital role in mitigating stampede risk during mass gatherings. Most studies reported measurable improvements in crowd flow indicators, such as reduced density peaks and shortened evacuation time, when interventions were applied in combination rather than in isolation. Quantitatively, simulation-based studies indicated reductions of 30%–40% in peak crowd density and 20%–35% in bottleneck duration under optimized management conditions.

**FIGURE 5:**
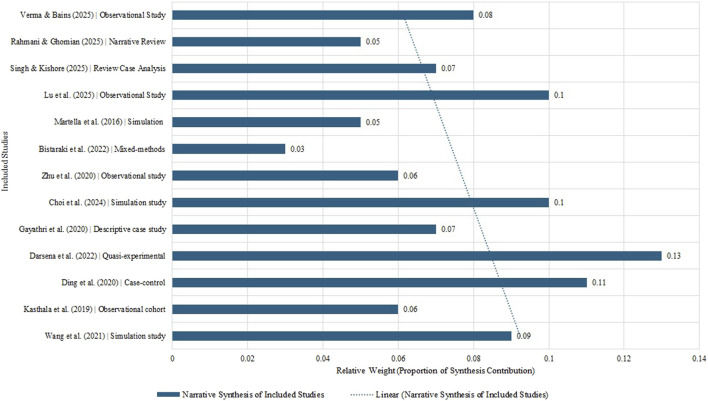
Narrative synthesis of included studies. Global, 2010–2025.

The weight synthesis graph (see Figure 5) summarizes the relative contribution of each study to the overall narrative synthesis [30]. The weight of each study was determined by its methodological robustness, data richness, and relevance to the review objectives. The studies that offer empirical or high-fidelity data, or clearer intervention-outcome relationships, have been given more weight than those that do not. The highest contributions came from Darsena et al. (0.13) and Ding et al. (0.11), followed closely by Lu et al. (0.10) and Choi et al. (0.10) [18]. High-impact field evaluations and frameworks, such as Verma and Bains (0.08) and Singh and Kishore (0.07), also provided substantial evidence to the synthesis [13, 16–18, 24, 25]. Comparative analysis revealed that not all studies contributed equally. Other studies provided useful context, but their quantitative evidence was limited [19, 23]. (Insert Figure 5 here).

## Discussion

This review examined the different kinds of evidence available on how stampedes can be prevented during large gatherings. Taken together, the findings suggest that no single intervention can fully prevent such incidents. Instead, prevention depends on a mix of coordinated, proactive measures that work together at multiple levels.

Beyond structural deficiencies, understanding the interaction between human behavior and environmental cues is important. Zhu et al. [20] used eye tracking to show that subjective ignorance of safety signage is a major barrier during evacuations. Their findings highlight a strong follower effect, where the presence of even one person moving in a particular direction can influence others, reducing the chances of choosing the correct exit by up to 30%. Furthermore, Martella et al. [19] argue that crowd members prioritize higher-level psychological needs, such as autonomy and social connection, in normal conditions, but shift abruptly to basic safety needs during a crisis. Martella et al. [19] also noted that variables such as age, gender, and former experience with an event significantly influence crowd behavior. For example, managing seated crowds in stadiums is inherently less complex than managing randomly moving crowds in transportation hubs or city centers. Additionally, groups such as families or friends require specific strategies to prevent separation, which is a major trigger for panic. Mass gatherings like marathons present unique “geographical spreads” and environmental challenges, where a sudden change in weather can trigger a mass, uncoordinated rush toward exits or transit points. Bistaraki and Georgiadis [23] further argue that the effectiveness of coordination depends on the history of interaction between agencies; those with established relationships communicate more efficiently than unfamiliar stakeholders. In their study, Bistaraki and Georgiadis [23] also found that interagency coordination is hindered by cultural differences and the use of specialized terminology that varies between police, medical, and voluntary services. To combat this, they advocate for the formal implementation of liaison officers during both the planning and implementation phases. These officers serve as vital links, ensuring shared situational awareness and preventing critical information from being lost in informal, personal communication channels or silos. This highlights that even the most robust technological systems are ineffective without established trust and synchronized roles among all stakeholders.

Similarly, Martella et al. [19] state that in normal situations, individuals in a crowd give importance to higher-level psychological needs such as autonomy and social connection. As seen in earlier work, planning, continuous monitoring, and clear communication are the main elements of crowd safety. Liang et al., for instance, noted that dense crowding and two-way movement were major contributors to the Seoul Itaewon tragedy, emphasizing the need for crowd flow management [29]. These case-specific observations build on earlier research that points to practical solutions, such as one-way pedestrian flow and timely crowd alerts. Furthermore, while disasters are often attributed to irrational crowd behavior, recent analysis suggests that underlying structural failures in high-density religious settings are the primary drivers of catastrophe, emphasizing that engineering integrity is as vital as behavioral management [25]. Likewise, Lu et al. observed that the nature of an event itself changes the level of risk, meaning that prevention measures should be adjusted to suit the context; spontaneous or unsupervised gatherings, for example, may need additional monitors and flexible barriers [24].

Technological interventions are becoming center of crowd safety, as most of the studies are showing their promising use in the real world. Simulation-based studies have shown the importance of using digital tools to help event organizers and managers run various test cases virtually. These “what-if” conditions, when run even before the event, help them understand how crowd flow, entry-exit pathways, and barrier placements can affect overcrowding. Choi et al. have shown that simulation-based systems are particularly capable of predicting density and bottleneck pressure points across various scenarios [18]. This could help decision-makers quantify potential risks and plan an event based on the calculated risk. This predictive modeling does not promise to replace the traditional safety measures completely; rather, it complements the crowd safety system. Similarly, Ha suggested that the use of high-end information and communication technologies, including artificial intelligence, should be an integral part of the safety framework for stampede prevention, thereby strengthening it by providing an early detection and response mechanism [27]. While simulation-based studies [18] identify density risks, newer perspectives suggest that social media sentiment analysis can predict the emotional state of a crowd, providing an earlier warning mechanism than physical sensors alone [14].

The study by Bistaraki and Georgiadis investigated how professionals perceive coordination during huge gatherings. It discussed the 2017 Athens Marathon, identifying three cohesive elements that play a crucial role in crowd management: (1) building a common bond of coordination among different agencies participating in the event; (2) conspicuously defining roles and responsibilities for security personnel to avoid mishap, confirming no confusion exists; and (3) deploying officers in each area for correct and speedy information sharing. Their findings emphasize that even with robust infrastructure and technology, crowd safety can still be at risk and break down [23]. It aligns with the emphasis of this review that, alongside engineering designs and digital support systems, people’s cooperation, trust, and synchronization are also needed to prevent stampedes.

Unlike the previous reviews, which mainly focused on reiterating the historically based or one-time events, this review tends to comprehensively focus on the systematic perspective, showing a vast variety of study contexts. It includes evidence from religious gatherings, entertainment or recreational events, and transportation sites to understand the conventions of crowd control and their application in real event settings. There were common measures that were consistent and effective across various settings, such as supervising the number of participants entering the area and ensuring clear visibility of exit routes, resulting in panic-free behavior and an unclogged, stress-free situation. This uniformity in protocol is more reliable to implement across a variety of events and, in a civic sense, helps escape the narrow passageways in critical conditions, thereby preventing stampedes [26].

Despite the above-noted preventive measures, several important research gaps persist and require prompt attention. There is a lack of real-world interventions on preventive strategies, leading to weak evidence. Mostly, existing research relied on observations or computer-based simulation modeling rather than experimental designs. Although simulation-based studies provide important predictive insights, their effectiveness in real-world settings remains constrained and uncertain due to the dynamic conditions and behavioral unpredictability inherent in live events. However, the field is moving toward large-scale validation; for instance, the successful management of 660 million devotees during the 2025 Maha Kumbh Mela serves as a benchmark, proving that tiered operational frameworks and Integrated Command Centers (ICC) can effectively maintain safety even in extreme-density scenarios [13].

There is a lack of standardized metrics, as across studies different metrics have been proposed or used. Some researchers used metrics such as crowd density and evacuation time, while others used perceived safety scores, which makes direct comparisons difficult. Hence, future researchers need a consensus on a common set of protocols to test the design’s robustness. Finally, there is minimal context on local and cultural factors that have been overlooked in the existing analyses. The major contributors to stampedes, such as social behaviors, localized crowd dynamics, communication patterns, and public attitudes toward preventive measures, are rarely investigated, even though they are direct impediments to the successful implementation of preventive measures against stampedes.

The findings of this review necessitate shifting from reactive crowd management to proactive, evidence-based policy frameworks. Policymakers who mandate the integration of engineering, operational, and technological controls must utilize a multi-modal regulatory approach. Specifically, national and local authorities should consider establishing mandatory safety auditing protocols for mass gatherings exceeding specific capacity thresholds. These protocols should adopt formalized paradigms, such as the five-stage risk reduction framework (decision, approval, assessment, integration, and planning), to replace reactive, *ad hoc* measures [25]. These audits should require event organizers to submit detailed crowd dynamics simulations and risk assessment plans before permit issuance, ensuring that infrastructure design (e.g., one-way flows, barrier placement) aligns with predicted crowd densities. Public health agencies and international bodies (e.g., WHO, ISO) should collaborate to develop a Core Outcome Set (COS) for crowd management research. Policies should formalize workforce certification and unified interagency command structures to reduce human error and crowd-related fatalities.

### Strengths and Limitations

The current review adhered to rigorous methodological standards, following PRISMA and PRISMA-S guidelines. A comprehensive search across several databases was conducted, and gray literature was included. Data extraction and quality assessment were conducted by multiple reviewers to analyse and assess quality, thereby minimizing error. By focusing on preventive and operational strategies (rather than descriptive incident reviews), this synthesis provides practical insights for stakeholders.

The majority of included studies were non-randomized or modeling-based, therefore limiting causal inference. Publication bias is possible, as successful interventions may be more frequently reported. Some relevant studies were omitted due to language and accessibility constraints. A relatively small number of studies met the inclusion criteria, reflecting the limited availability of high-quality empirical research in this field, which may constrain the generalizability of findings. The heterogeneity of settings and outcomes precluded meta-analysis; hence, our results are primarily a qualitative synthesis. Due to limited empirical data, simulation-based evidence alone is insufficient to determine the effectiveness of measures unless they undergo implementation and field evaluation before policy adoption.

### Conclusion

The findings of this systematic review demonstrate that stampede at mass public gatherings are largely preventable through the implementation of integrated, evidence-based strategies. Rather than relying on isolated interventions, effective crowd safety depends on the synergistic application of engineering controls, operational planning, technology-enabled monitoring, and clear communication protocols. There are key measures consistently associated with improved outcomes, such as establishing one-way pedestrian flows, enforcing density caps, deploying trained personnel at critical bottlenecks, and using real-time surveillance systems to detect emerging hazards. Event organizers, public health authorities, and emergency response agencies should collaborate during the pre-event planning phase to develop context-specific, multimodal safety frameworks. These frameworks should also prioritize practical, scalable actions, such as removing physical bottlenecks, improving signage visibility, and standardizing emergency communication, while remaining adaptable to the unique dynamics of different gathering types.

Future research should prioritize the development of standardized measurement protocols and the rigorous field testing of preventive interventions across diverse cultural and geographic settings. By addressing these gaps and institutionalizing proactive, multi-sectoral approaches, stakeholders can move decisively toward minimizing preventable injuries and fatalities at large public gatherings worldwide.

## Data Availability

All data generated or analysed during this study are included in this article and Supplementary Materials S1–S8.
