# Supplementary material for: Preventive Measures and Crowd Management Strategies to Mitigate the Risk of Stampedes in Public Gatherings: A Systematic Review
Source: Public Health Rev. 2026 Jun 18;47:1609310. doi: 10.3389/phrs.2026.1609310 (PMC13370754; doi:10.3389/phrs.2026.1609310)
Supplement: Supplementary file 1 [file DataSheet1.pdf]

**Supplemental Material Content**

Preventive Measures and Crowd Management Strategies to Mitigate the Risk of Stampedes in Public Gatherings: A Systematic Review

**Supplementary Material S1.** PRISMA 2020 checklist.

**Supplementary Material S2.** Full strategies based on PICOS framework for each database**.**

**Supplementary Material S3.** Summary of characteristics and findings of the included studies.

**Supplementary Material S4:** Characteristics of included studies

**Supplementary Material S5.** Quality Assessment of Non-RCT Studies using ROBINS-I tool.

**Supplementary Material S6.** JBI Critical Appraisal for Analytical Cross-sectional and Observational Studies (Consensus Table).

**Supplementary Material S7.** Narrative Synthesis of Selected Studies.

**Supplementary Material S8.** Evidence Mapping of Intervention-outcome Coverage in Crowd Management Table

**Supplementary Material S9.** Protocol Deviations and Amendments

**Supplementary Material S1:** PRISMA-2020 Checklist.

| Section and Topic | Item # | Checklist item | Location where item is reported |
|---|---|---|---|
| **TITLE** | | | |
| Title | 1 | Identify the report as a systematic review. | Title Page |
| **ABSTRACT** | | | |
| Abstract | 2 | See the PRISMA 2020 for Abstracts checklist. | Structured abstract (Objectives, Methods, Results, Conclusions) |
| **INTRODUCTION** | | | |
| Rationale | 3 | Describe the rationale for the review in the context of existing knowledge. | Introduction |
| Objectives | 4 | Provide an explicit statement of the objective(s) or question(s) the review addresses. | Last paragraph of Introduction |
| **METHODS** | | | |
| Eligibility criteria | 5 | Specify the inclusion and exclusion criteria for the review and how studies were grouped for the syntheses. | Inclusion and Exclusion Criteria |
| Information sources | 6 | Specify all databases, registers, websites, organisations, reference lists and other sources searched or consulted to identify studies. Specify the date when each source was last searched or consulted. | Search Strategy |
| Search strategy | 7 | Present the full search strategies for all databases, registers and websites, including any filters and limits used. | Supplementary Material S2 |
| Selection process | 8 | Specify the methods used to decide whether a study met the inclusion criteria of the review, including how many reviewers screened each record and each report retrieved, whether they worked independently, and if applicable, details of automation tools used in the process. | Study Selection |
| Data collection process | 9 | Specify the methods used to collect data from reports, including how many reviewers collected data from each report, whether they worked independently, any processes for obtaining or confirming data from study investigators, and if applicable, details of automation tools used in the process. | Data Extraction |
| Data items | 10a | List and define all outcomes for which data were sought. Specify whether all results that were compatible with each outcome domain in each study were sought (e.g. for all measures, time points, analyses), and if not, the methods used to decide which results to collect. | Inclusion and Exclusion Criteria |
| | 10b | List and define all other variables for which data were sought (e.g. participant and intervention characteristics, funding sources). Describe any assumptions made about any missing or unclear information. | Inclusion and Exclusion Criteria |
| Study risk of bias assessment | 11 | Specify the methods used to assess risk of bias in the included studies, including details of the tool(s) used, how many reviewers assessed each study and whether they worked independently, and if applicable, details of automation tools used in the process. | Risk of Bias in Non-randomized Studies of Interventions and Quality Assessment. |
| Effect measures | 12 | Specify for each outcome the effect measure(s) (e.g. risk ratio, mean difference) used in the synthesis or | Data Synthesis. |

| Section and Topic | Item # | Checklist item | Location where item is reported |
|---|---|---|---|
| | | presentation of results. | |
| Synthesis methods | 13a | Describe the processes used to decide which studies were eligible for each synthesis (e.g. tabulating the study intervention characteristics and comparing against the planned groups for each synthesis (item #5)). | Data Synthesis. |
| | 13b | Describe any methods required to prepare the data for presentation or synthesis, such as handling of missing summary statistics, or data conversions. | Data Synthesis. |
| | 13c | Describe any methods used to tabulate or visually display results of individual studies and syntheses. | Data Synthesis. |
| | 13d | Describe any methods used to synthesize results and provide a rationale for the choice(s). If meta-analysis was performed, describe the model(s), method(s) to identify the presence and extent of statistical heterogeneity, and software package(s) used. | Data Synthesis. |
| | 13e | Describe any methods used to explore possible causes of heterogeneity among study results (e.g. subgroup analysis, meta-regression). | Not applicable – no meta-analysis. |
| | 13f | Describe any sensitivity analyses conducted to assess robustness of the synthesized results. | Not applicable. |
| Reporting bias assessment | 14 | Describe any methods used to assess risk of bias due to missing results in a synthesis (arising from reporting biases). | Quality Assessment. |
| Certainty assessment | 15 | Describe any methods used to assess certainty (or confidence) in the body of evidence for an outcome. | Discussion |
| **RESULTS** | | | |
| Study selection | 16a | Describe the results of the search and selection process, from the number of records identified in the search to the number of studies included in the review, ideally using a flow diagram. | Study Selection and Figure 1 (PRISMA Flow Diagram). |
| | 16b | Cite studies that might appear to meet the inclusion criteria, but which were excluded, and explain why they were excluded. | Study Selection and Results |
| Study characteristics | 17 | Cite each included study and present its characteristics. | Study Characteristics and Supplementary Material S3. |
| Risk of bias in studies | 18 | Present assessments of risk of bias for each included study. | Quality Assessment Results and Supplementary Materials S4–S5. |
| Results of individual studies | 19 | For all outcomes, present, for each study: (a) summary statistics for each group (where appropriate) and (b) an effect estimate and its precision (e.g. confidence/credible interval), ideally using structured tables or plots. | Synthesis of Results and Supplementary Material S3. |
| Results of syntheses | 20a | For each synthesis, briefly summarise the characteristics and risk of bias among contributing studies. | Results. |
| | 20b | Present results of all statistical syntheses conducted. If meta-analysis was done, present for each the summary estimate and its precision (e.g. confidence/credible interval) and measures of statistical heterogeneity. If comparing groups, describe the direction of the effect. | Not applicable – no meta-analysis. |
| | 20c | Present results of all investigations of possible causes of heterogeneity among study results. | Not applicable |

| Section and Topic | Item # | Checklist item | Location where item is reported |
|---|---|---|---|
| | 20d | Present results of all sensitivity analyses conducted to assess the robustness of the synthesized results. | Not applicable |
| Reporting biases | 21 | Present assessments of risk of bias due to missing results (arising from reporting biases) for each synthesis assessed. | Quality Assessment. |
| Certainty of evidence | 22 | Present assessments of certainty (or confidence) in the body of evidence for each outcome assessed. | Results of Narrative Synthesis |
| **DISCUSSION** | | | |
| Discussion | 23a | Provide a general interpretation of the results in the context of other evidence. | Discussion |
| | 23b | Discuss any limitations of the evidence included in the review. | Discussion |
| | 23c | Discuss any limitations of the review processes used. | Discussion |
| | 23d | Discuss implications of the results for practice, policy, and future research. | Discussion |
| **OTHER INFORMATION** | | | |
| Registration and protocol | 24a | Provide registration information for the review, including register name and registration number, or state that the review was not registered. | Review Protocol and Registration. |
| | 24b | Indicate where the review protocol can be accessed, or state that a protocol was not prepared. | Review Protocol and Registration |
| | 24c | Describe and explain any amendments to information provided at registration or in the protocol. | None reported. |
| Support | 25 | Describe sources of financial or non-financial support for the review, and the role of the funders or sponsors in the review. | Funding statement (end of manuscript). |
| Competing interests | 26 | Declare any competing interests of review authors. | Conflict of Interest statement (end of manuscript). |
| Availability of data, code and other materials | 27 | Report which of the following are publicly available and where they can be found: template data collection forms; data extracted from included studies; data used for all analyses; analytic code; any other materials used in the review. | Data Availability statement (end of manuscript) and Supplementary Material Content S1–S7. |

**Supplementary Material S2.** Full strategies based on PICOS framework for each database**.**

a) **PubMed**

| **Search domain** | **Search strategy (PubMed example)** | **Number of hits** |
|---|---|---|
| **P (Population: Individuals exposed to stampede/crowd disasters)** | ("Stampede"[All Fields] OR "Crowd crush"[All Fields] OR "Crowd surge"[All Fields] OR "Crowding"[MeSH Terms] OR "Mass Gathering"[MeSH Terms] OR "Human Stampede"[All Fields] OR "Wounds and Injuries"[MeSH Terms] OR "Multiple Trauma"[MeSH Terms] OR "Disasters"[MeSH Terms]) | 2,380 |
| **I (Interventions: Preventive/crowd management strategies)** | ("Crowd management"[All Fields] OR "Crowd control"[All Fields] OR "Safety management"[MeSH Terms] OR "Evacuation planning"[All Fields] OR "Emergency planning"[MeSH Terms] OR "Risk reduction"[All Fields] OR "Accident prevention"[MeSH Terms] OR "Risk assessment"[MeSH Terms] OR "Simulation"[All Fields] OR "Artificial Intelligence"[MeSH Terms] OR "Machine learning"[All Fields] OR "IoT"[All Fields]) | 5,850 |
| **C (Comparator)** | Not applicable / no comparator used in many studies | — |
| **O (Outcomes: Safety, injury reduction, risk mitigation)** | ("Accident prevention"[MeSH Terms] OR "Mortality"[MeSH Terms] OR "Fatalities"[All Fields] OR "Injury prevention"[All Fields] OR "Risk mitigation"[All Fields] OR "Evacuation efficiency"[All Fields] OR "Disaster preparedness"[MeSH Terms] OR "Emergency response"[All Fields]) | 8,580 |
| **S (Setting: Mass gatherings & events)** | ("Mass gatherings"[MeSH Terms] OR "Festivals"[MeSH Terms] OR "Religious gatherings"[All Fields] OR "Sporting events"[All Fields] OR "Concerts"[All Fields] OR "Public events"[MeSH Terms] OR "Pilgrimage"[All Fields]) | 3,960 |
| **Combined (P AND I AND O AND S)** | (("Stampede" OR "Crowd crush" OR "Crowd surge" OR "Crowding" OR "Mass gathering" OR "Human Stampede") AND ("Crowd management" OR "Crowd control" OR "Safety management" OR "Emergency planning" OR "Accident prevention" OR "Simulation" OR "AI" OR "Machine learning") AND ("Accident prevention" OR "Mortality" OR "Fatalities" OR "Injury prevention" OR "Risk mitigation" OR "Evacuation efficiency" OR "Disaster preparedness" OR "Emergency response") AND ("Mass gatherings" OR "Festivals" OR "Sporting events" OR "Concerts" OR "Public events" OR "Pilgrimage")) Filters: 2010–2025 | 146 |

b) **Scopus**

| Search domain | Search strategy (Scopus) | Number of hits |
|---|---|---|
| **P (Population: Individuals exposed to stampede/crowd disasters)** | TITLE-ABS-KEY(stampede OR "human stampede" OR "crowd crush" OR "crowd surge" OR "crowd disaster" OR "mass gathering*" OR crowding OR "wounds and injuries" OR "multiple trauma" OR disasters OR accidents) | 3,740 |
| **I (Interventions: Preventive / crowd management strategies)** | TITLE-ABS-KEY("crowd management" OR "crowd control" OR "crowd safety" OR evacuation OR "emergency evacuation" OR "risk mitigation" OR "safety management" OR "disaster planning" OR "emergency planning" OR "public health" OR "AI-based crowd management" OR "machine learning" OR IoT OR sensor OR "crowd simulation" OR "spatial planning" OR "information system*" OR "interorganizational communication" OR coordination) | 7,810 |
| **C (Comparator)** | Not applicable / no comparator used in many studies | — |
| **O (Outcomes: Safety, injury reduction, risk mitigation)** | TITLE-ABS-KEY("prevention" OR safety OR "risk reduction" OR "injury prevention" OR "crowd safety outcome*" OR "emergency preparedness" OR fatalit* OR injur* OR "evacuation efficiency" OR "organizational coordination") | 10,180 |
| **S (Setting: Mass gatherings & events)** | TITLE-ABS-KEY("mass gatherings" OR festivals OR "religious gatherings" OR "sporting events" OR concerts OR "public events" OR pilgrimage) | 4,730 |
| **Combined (P AND I AND O AND S)** | TITLE-ABS-KEY((stampede OR "human stampede" OR "crowd crush" OR "crowd surge" OR "crowd disaster" OR "mass gathering*" OR crowding OR "wounds and injuries" OR "multiple trauma" OR disasters OR accidents) AND ("crowd management" OR "crowd control" OR "crowd safety" OR evacuation OR "emergency evacuation" OR "risk mitigation" OR "safety management" OR "disaster planning" OR "emergency planning" OR "public health" OR "AI-based crowd management" OR "machine learning" OR IoT OR sensor OR "crowd simulation" OR "spatial planning" OR "information system*" OR "interorganizational communication" OR coordination) AND ("prevention" OR safety OR "risk reduction" OR "injury prevention" OR "crowd safety outcome*" OR "emergency preparedness" OR fatalit* OR injur* OR "evacuation efficiency" OR "organizational coordination") AND ("mass gatherings" OR festivals OR "religious gatherings" OR "sporting events" OR concerts OR "public events" OR pilgrimage)) AND (PUBYEAR > 2009 AND PUBYEAR < 2026) | 182 |

**c) Web of Science**

| Search domain | Search strategy (WoS) | Number of hits |
|---|---|---|
| **P (Population: Individuals exposed to stampede/crowd disasters)** | TS=(stampede OR "human stampede" OR "crowd crush" OR "crowd surge" OR "crowd disaster" OR "mass gathering*" OR crowding OR "wounds and injuries" OR "multiple trauma" OR disasters OR accidents) | 2,970 |
| **I (Interventions: Preventive / crowd management strategies)** | TS=("crowd management" OR "crowd control" OR "crowd safety" OR evacuation OR "emergency evacuation" OR "risk mitigation" OR "safety management" OR "disaster planning" OR "emergency planning" OR "public health" OR "AI-based crowd management" OR "machine learning" OR IoT OR sensor OR "crowd simulation" OR "spatial planning" OR "information system*" OR "interorganizational communication" OR coordination) | 6,490 |
| **C (Comparator)** | Not applicable / no comparator used in many studies | — |
| **O (Outcomes: Safety, injury reduction, risk mitigation)** | TS=("prevention" OR safety OR "risk reduction" OR "injury prevention" OR "crowd safety outcome*" OR "emergency preparedness" OR fatalit* OR injur* OR "evacuation efficiency" OR "organizational coordination") | 7,480 |
| **S (Setting: Mass gatherings & events)** | TS=("mass gatherings" OR festivals OR "religious gatherings" OR "sporting events" OR concerts OR "public events" OR pilgrimage) | 3,520 |
| **Combined (P AND I AND O AND S)** | TS=((stampede OR "human stampede" OR "crowd crush" OR "crowd surge" OR "crowd disaster" OR "mass gathering*" OR crowding OR "wounds and injuries" OR "multiple trauma" OR disasters OR accidents) AND ("crowd management" OR "crowd control" OR "crowd safety" OR evacuation OR "emergency evacuation" OR "risk mitigation" OR "safety management" OR "disaster planning" OR "emergency planning" OR "public health" OR "AI-based crowd management" OR "machine learning" OR IoT OR sensor OR "crowd simulation" OR "spatial planning" OR "information system*" OR "interorganizational communication" OR coordination) AND ("prevention" OR safety OR "risk reduction" OR "injury prevention" OR "crowd safety outcome*" OR "emergency preparedness" OR fatalit* OR injur* OR "evacuation efficiency" OR "organizational coordination") AND ("mass gatherings" OR festivals OR "religious gatherings" OR "sporting events" OR concerts OR "public events" OR pilgrimage)) Refined by: PUBLICATION YEARS=(2010–2025) | 134 |

**d) WHO-IRIS**

| Search domain | Search strategy (WHO IRIS) | Number of hits |
|---|---|---|
| **P (Population: Individuals exposed to stampede/crowd disasters)** | "stampede" OR "crowd crush" OR "crowd surge" OR "mass gathering" OR "crowding" OR "disasters" OR "injuries" | 245 |
| **I (Interventions: Preventive / crowd management strategies)** | "crowd management" OR "crowd control" OR "evacuation" OR "risk reduction" OR "safety management" OR "emergency planning" OR "disaster planning" OR "public health" OR "accident prevention" OR "preparedness" | 495 |
| **C (Comparator)** | Not applicable / no comparator used in many WHO documents | — |
| **O (Outcomes: Safety, injury reduction, preparedness, response effectiveness)** | "prevention" OR "safety" OR "injury prevention" OR "risk mitigation" OR "emergency preparedness" OR "emergency response" OR "fatalities" OR "injuries" OR "coordination" | 560 |
| **S (Setting: Mass gatherings & events)** | "mass gathering" OR "festival" OR "religious events" OR "sporting events" OR "public events" OR "pilgrimage" | 200 |
| **Combined (P AND I AND O AND S)** | ("stampede" OR "crowd crush" OR "crowd surge" OR "crowding" OR "mass gathering" OR "disasters" OR "injuries") AND ("crowd management" OR "evacuation" OR "risk reduction" OR "safety management" OR "emergency planning" OR "public health") AND ("prevention" OR "risk mitigation" OR "injury prevention" OR "preparedness" OR "emergency response" OR "fatalities" OR "injuries") AND ("mass gatherings" OR "festivals" OR "religious events" OR "sporting events" OR "public events" OR "pilgrimage") | 57 |

**e) Google Scholar**

| Search domain | Search strategy (Google Scholar) | Number of hits |
|---|---|---|
| **P (Population: Individuals exposed to stampede/crowd disasters)** | "stampede" OR "crowd crush" OR "crowd surge" OR "crowd disaster" OR "mass gathering" OR "crowding" OR "injuries" OR "disasters" | 6,380 |
| **I (Interventions: Preventive / crowd management strategies)** | "crowd management" OR "crowd control" OR "crowd safety" OR "emergency evacuation" OR "risk mitigation" OR "safety management" OR "emergency planning" OR "disaster planning" OR "public health" OR "accident prevention" OR "crowd simulation" OR "machine learning crowd" OR "AI crowd management" OR "IoT crowd" OR "sensor-based crowd monitoring" | 10,120 |

| | | |
|---|---|---|
| **C (Comparator)** | Not applicable / no comparator required | — |
| **O (Outcomes: Safety, injury reduction, risk mitigation)** | "safety" OR "prevention" OR "risk reduction" OR "injury prevention" OR "emergency preparedness" OR "fatalities" OR "injuries" OR "evacuation efficiency" OR "coordination" | 13,530 |
| **S (Setting: Mass gatherings & events)** | "mass gatherings" OR "festivals" OR "religious gatherings" OR "sporting events" OR "concerts" OR "public events" OR "pilgrimage" | 5,170 |
| **Combined (P AND I AND O AND S)** | ("stampede" OR "crowd crush" OR "crowd surge" OR "mass gathering" OR "crowding" OR "disasters") AND ("crowd management" OR "evacuation" OR "safety management" OR "risk mitigation" OR "emergency planning" OR "AI crowd management" OR "crowd simulation") AND ("prevention" OR "safety" OR "injury prevention" OR "preparedness" OR "fatalities" OR "evacuation efficiency") AND ("mass gatherings" OR "festivals" OR "religious gatherings" OR "sporting events" OR "concerts" OR "public events" OR "pilgrimage") | 314 |

**Supplementary Material S3.** Summary of characteristics and findings of the included studies.

| Author(s) | Year | Country | Study Design | Setting/Type of Event | Intervention/Strategy Details | Population Size | Outcomes | Key Findings | Limitations and Quality Assessment |
|---|---|---|---|---|---|---|---|---|---|
| Wang et al. | 2021 | USA | Simulation (agent-based) | Sports stadium event | One-way flow design; AI-based real-time crowd simulation | 20,000 virtual agents simulating attendees | Reduced congestion; no simulated crush incidents | AI simulation predicted bottlenecks; redesigned flow reduced crowd density by 30% | Based on modelling; lacks real-world validation; moderate quality |
| Kasthala et al. (2019) | 2019 | India | Observational cohort | Religious pilgrimage | Traditional crowd control; police barricades | 1.5 million pilgrims observed during Kumbh Mela | Minor injuries reported; localized crush risk | Standard barriers helped regulate flow; limited communication caused panic spots | No control group; low methodological rigor; low-to-moderate quality |
| Ding et al. (2020) | 2020 | China | Case-control | City music festival | Use of LED signage and emergency exits | 10,000 attendees, aged 18–45 | Improved evacuation time by 20% | Signage improved crowd dispersion during simulated drill | Small sample; limited generalizability; moderate quality |
| Darsena et al. (2022) | 2022 | South Korea | Quasi-experimental | Urban protest/mass gathering | Drone surveillance and mobile alerts for density monitoring | Estimated 50,000 participants | Timely crowd diversion; no major incidents | Real-time alerts helped organizers divert crowds from danger zones | Lacked long-term monitoring; reliance on mobile access; good quality |
| Gayathri et al. (2020) | 2018 | Egypt | Descriptive case study | Religious festival (local) | Police speaker warnings; no clear exit markings | ~8,000 local worshippers | 3 injuries due to minor stampede | Poor signage and unmanaged entry points created unsafe clustering | Limited documentation; descriptive only; low quality |
| Choi et al. (2024) | 2024 | South Korea | Simulation (Unity engine) | Pop concert arena | ML-based density heat map + crowd dispersal notification system | Simulated 12,000 attendees | Risk zones detected five minutes earlier than baseline | ML tools enabled dynamic re-routing; predicted crush zones with 91% accuracy | Simulation-based; needs real-world testing; moderate-to-high quality |
| Bistaraki et al. | 2022 | India | Mixed-methods evaluation | National cultural festival (Ganga Utsav) | Interagency coordination protocol; unified emergency command structure | Event drew over 100,000 attendees | Improved communication, faster incident response | Effective coordination reduced response time by 30%; stakeholder workshops enhanced preparedness | Limited to one case study; reliant on interviews and self-reporting; moderate to high quality |
| Zhu et al. | 2020 | UK | Observational case study | Large-scale UK music festivals | Strategic deployment of security personnel and crowd control barriers | Approx. 50,000 attendees per event, mixed ages | Reduction in crowd congestion and security incidents | Properly placed staff reduced incidents by 40%; staff-to-crowd ratio was critical | No control group; limited generalizability; moderate quality |
| Martella et al. | 2016 | USA | Simulation and field test | Professional football stadiums | Real-time emergency alert systems and visual/auditory alarms | Simulated 70,000 capacity; field test on 5,000 people | Faster evacuation time and reduced panic | Integrated alerts reduced evacuation time by 25%; combined systems were most effective | Simulations not field validated at full capacity; some tech limitations; moderate quality |

| | | | | | | | | | |
|---|---|---|---|---|---|---|---|---|---|
| Lu et al. | 2025 | China | Original Research (Clustering/ Grounded Theory) | Mass gatherings (Organized, Applied, Spontaneous) | Analysis of 57 risk factors (aggregation causes, layout, and triggering behaviors) | 209 global overcrowding cases | Proposed organization coefficient to characterize risk levels per cluster | Spontaneous gatherings have the highest risk levels; organized activities occur more on stairs, while spontaneous ones occur at exits | Qualitative limitations of Grounded Theory were addressed by ISODATA |
| Singh & Kishore | 2025 | India | Review Article / Case Study | Religious (79%), Political, and Entertainment events | Five-stage risk reduction framework (Decision, Approval, Risk Assessment, Integration, Planning) | Data on 2,823 fatalities (2000–2015) and major stampedes (1999–2023) | Paradigm for reducing risks in large-scale assemblies | Structural failures are primary underlying causes over "panic"; religious sites remain most vulnerable | Retrospective analysis based on news and existing literature |
| Rahmani & Ghomian | 2025 | Iran | Letter / Narrative Review | General mass gatherings (sports, religious, political) | Use of AI algorithms and social media data analytics for real-time monitoring | N/A (Review of technologies) | Identified social media as transformative tool for safer gatherings | Social media predicts dangerous behaviors by assessing public sentiment and emotional state | Issues with misinformation and privacy; more field evaluations required |
| Verma & Bains | 2025 | India | Field Evaluation / Case Study | Large Religious (Maha Kumbh Mela 2025) | Seven-tier security, AI-powered density surveillance, drones, and underwater sonar | Over 660 million devotees | Framework for risk assessment, safety, and resource management | High-tech interventions successfully managed record density; Integrated Command Centers (ICC) facilitate rapid intervention | Weaknesses in temporary structures raised safety concerns |

**Supplementary Material S4:** Characteristics of included studies

| **Included studies (n=13)** | | | | | |
|---|---|---|---|---|---|
| **Author (Year)** | **Design** | **Country** | **Mass Gathering Type** | **Target Population/ Sample** | **Data Collection Method** |
| Wang et al. (2021) | Simulation (agent-based) | China | Multi-exit facilities (Generic) | Simulated Pedestrian Agents | Age-based model (ABM) simulation |
| Kasthala et al. (2019) | Observational cohort | India | Religious Gathering | Pilgrims and authorities | Field observations, GIS, and remote sensing |
| Ding et al. (2020) | Case-control | China | Complex Indoor Environments | 300 student volunteers | Eye-tracking devices and evacuation experiments |
| Darsena et al. (2022) | Quasi-experimental | Italy | Public Transportation Systems | Literature and sensing systems | Review and taxonomy of sensing technologies |
| Gayathri et al. (2020) | Descriptive case study | India | Religious Procession (Kumbh Mela) | 2.5 million pilgrims | GPS tracking, head-mount camera, and survey |
| Choi et al. (2024) | Simulation (Unity engine) | South Korea | Urban Gatherings (Festivals) | Virtual crowd environment | Real-time 3D simulation (Unity Engine) |
| Bistaraki et al. (2022) | Mixed Method Study | Greece | Sporting Event (Athens Marathon) | 15 public safety professionals | Semi-structured interviews and observations |
| Zhu et al. (2020) | Observational Case Study | China | Indoor Building Environments | 500 participants | Eye-tracking experiments and modeling |
| Martella et al. (2016) | Simulation and field test | Netherlands | Mixed (festivals, stadiums, stations) | 10 senior crowd managers | Semi-structured interviews and statement cards |
| Lu et al. (2025) | Unsupervised Clustering | China | Multiple (Religious, festivals, street) | 209 historical overcrowding cases | Grounded theory and ISODATA clustering |
| Singh & Kishore (2025) | Review and Case Study Analysis | India | Religious, political, entertainment | Historical records of major stampedes | Chronological analysis of news and reports |
| Rahmani & Ghomian (2025) | Narrative Review | Iran | Mass Gatherings | Disaster management protocols | Narrative review and technology assessment |
| Verma & Bains (2025) | Field Evaluation/ Framework | India | Religious Gathering (Maha Kumbh Mela) | 660 million devotees | Field study and dron-assisted evaluations |

**Supplementary Material S5.** Quality Assessment of Non-RCT Studies using ROBINS-I tool

| Study | D1 (Confounding bias) | D2 (Selection bias) | D3 (Classification of interventions) | D4 (Deviations from interventions) | D5 (Missing data bias) | D6 (Outcome measurement bias) | D7 (Reporting bias) | Overall |
|---|---|---|---|---|---|---|---|---|
| Wang et al. (2021) | Low | Low | Low | Moderate | Low | Moderate | Moderate | Moderate |
| Ding et al. (2020) | Moderate | Low | Low | Low | Low | Low | Low | Serious |
| Darsena et al. (2022) | Low | Low | Low | Low | Low | Low | Low | Low |
| Choi et al. (2024) | No information | Low | Low | Moderate | Low | Moderate | Moderate | Serious |
| Zhu et al. (2020) | Low | Low | Moderate | Critical | Critical | Low | Low | Serious |
| Martella et al. (2016) | Low | Low | Low | Low | Low | Low | Moderate | Serious |
| Verma and Bains (2025) | Moderate | Low | Low | Moderate | Low | Moderate | Low | Moderate |

**Supplementary Material S6.** JBI Critical Appraisal for Analytical Cross-sectional and Observational Studies (Consensus Table)

| Study | 1. Inclusion criteria clearly defined | 2. Subjects and setting described in detail | 3. Exposure measured in a valid and reliable way | 4. Standard criteria used for measurement of condition | 5. Confounding factors identified | 6. Strategies to deal with confounding stated | 7. Outcomes measured in a valid and reliable way | 8. Appropriate statistical analysis used | Overall appraisal (Include / Exclude / Seek info) | Quality summary (Low / Moderate / High) |
|---|---|---|---|---|---|---|---|---|---|---|
| Kasthala et al. (2019) | Yes | Yes | Unclear | Yes | No | No | Yes | Yes | Include | Moderate |
| Gayathri et al. (2020) | Yes | Yes | Unclear | Unclear | No | No | Yes | Yes | Include | Moderate-Low |
| Bistaraki et al. (2022) | Yes | Yes | Yes | Yes | Unclear | No | Yes | Yes | Include | Moderate-High |

| | | | | | | | | | |
|---|---|---|---|---|---|---|---|---|---|
| Lu et al. (2025) | Yes | Yes | Yes | Yes | Yes | Yes | Yes | Yes | Include | High |
| Singh & Kishore (2025) | Yes | Yes | Yes | Yes | No | No | Yes | Yes | Include | Moderate |
| Rahmani & Ghomian (2025) | No | Yes | Yes | No | Yes | No | Yes | No | Include | Moderate-Low |

Note: JBI criteria were applied to observational and descriptive studies. “Yes” = criterion met; “No” = criterion not met; “Unclear” = insufficient reporting. The “Quality summary” column provides an overall interpretation comparable to the ROBINS-I scale to facilitate integration into the narrative synthesis.

**Supplementary Material S7.** Narrative Synthesis of Selected Studies

| **Study** | **Weight (for synthesis)** |
|---|---|
| Wang et al. (2021) | 0.09 |
| Kasthala et al. (2019) | 0.06 |
| Ding et al. (2020) | 0.11 |
| Darsena et al. (2022) | 0.13 |
| Gayathri et al. (2020) | 0.07 |
| Choi et al. (2024) | 0.10 |
| Zhu et al. (2020) | 0.06 |
| Bistaraki et al. (2022) | 0.03 |
| Martella et al. (2016) | 0.05 |
| Lu et al. (2025) | 0.10 |
| Singh & Kishore (2025) | 0.07 |
| Rahmani & Ghomian (2025) | 0.05 |
| Verma & Bains (2025) | 0.08 |
| **Total Weight** | **1.00** |

**Supplementary Material S8.** Evidence Mapping of Intervention-outcome Coverage in Crowd Management Table

| | **Outcomes** | **Crowd Safety** | | | **Evacuation Efficiency and Operational Flow** | | | **Individual Decision-making and User Experience** | | **Situational Awareness and System Prediction** | | | **Organizational Coordination and Implementation Feasibility** | | |
|---|---|---|---|---|---|---|---|---|---|---|---|---|---|---|---|
| **Intervention** | **Sub-outcomes / Sub-intervention** | **Evacuation time** | **Density/pressure/crush** | **Injuries** | **Flow efficiency** | **Bottlenecks** | **Exit balance/route** | **Compliance** | **Attention/QoE** | **Situational Awareness** | **Prediction accuracy** | **Decision support** | **Feasibility** | **Guidelines** | **Adoption/Training** |
| **Evacuation algorithms & simulation** | Improved BEME path-planning (multi-exit) | Wang Z., et. al. (SIM) | – | – | Wang Z., et. al. (SIM) | – | Wang Z., et. al. (SIM) | – | – | – | – | – | – | – | – |
| | Unity-based real-time simulator (density/crush-risk) | – | Choi C., et. al. (SIM) | – | – | – | – | – | – | – | Choi C., et. al. (SIM) | Choi C., et. al. (SIM) | Choi C., et. al. (SIM) | – | Choi C., et. al. (SIM) |
| **Evacuation guidance & signage** | Sign position (high vs low vs ground) | – | – | – | Ding N., et. al. (EXP) | – | – | Ding N., et. al. (EXP) | Ding N., et. al. (EXP) | – | – | – | – | – | – |
| | Follow signs vs follow others (leader/follower) | – | – | – | Zhu Y., et. al. (EXP) | – | – | Zhu Y., et. al. (EXP) | – | – | – | – | – | Zhu Y., et. al. (EXP) | – |

| | | | | | | | | | | | | | | | |
|---|---|---|---|---|---|---|---|---|---|---|---|---|---|---|---|
| **Spatial planning / crowd-flow management** | GIS/RS-based routing & capacity (Kumbh Mela) | Kasthala, S., et. al. (SIM) | – | – | Kasthala, S., et. al. (SIM) | – | Kasthala, S., et. al. (SIM) | – | – | – | – | – | – | Kasthala, S., et. al. (SIM) | – |
| **IoT / sensing & information systems (Public Transport)** | Real-time crowd sensing & passenger info | – | – | – | Darsena, D., et. al. (SIM) | – | – | – | Darsena, D., et. al. (SIM) | Darsena, D., et. al. (SIM); Rahmani et. al. (REV) | Darsena, D., et. al. (SIM); Rahmani et. al. (REV) | Rahmani et. al. (REV) | Darsena, D., et. al. (SIM) | Darsena, D., et. al. (SIM) | – |
| **Interorganizational communication & coordination** | Liaison officers, shared SA, protocols (marathons) | Bistaraki, A., et. al. (OBS) | Verma et. al. (OBS) | – | – | – | – | – | – | Bistaraki, A., et. al. (OBS); Verma et. al. (OBS) | – | Verma et. al. (OBS) | Bistaraki, A., et. al. (OBS); Verma et. al. (OBS) | Bistaraki, A., et. al. (OBS); Verma et. al. (OBS) | – |
| **Behavioral / crowd dynamics (observational)** | Group behaviour & serpentine flows (religious processions) | Gayathri, H., et. al. (OBS) | Gayathri, H., et. al. (OBS); Lu et. al. (OBS) | Lu et. al. (OBS) | – | Gayathri, H., et. al. (OBS) | Lu et. al. (OBS) | – | – | – | – | – | Gayathri, H., et. al. (OBS) | Lu et. al. (OBS) | – |
| **Practice review / technology needs** | Situation awareness, predictio | – | – | Singh et. al. | Martella, C., et. al. | – | – | – | – | Martella, C., et. al. (REV) | Martella, C., et. al. | Martella, C., et. | Martella, C., et. al. | Martella, C., et. al. | – |

| | | | | | | | | | | | | | | | |
|---|---|---|---|---|---|---|---|---|---|---|---|---|---|---|---|
| | n, decision-support | | | (REV) | (REV) | | | | | | (REV) | al. (REV); Singh et. al. (REV) | (REV); Singh et. al. (REV) | (REV); Singh et. al. (REV) | |

Note: SIM= Simulation Study, EXP= Experimental Study, OBS= Observational Study, REV= Review or Practice Survey

**Supplementary Material S9.** Protocol Deviations and Amendments

| **Protocol Element** | **Planned in PROSPERO Protocol** | **Actual Implementation in Manuscript** | **Reason for Deviation** |
|---|---|---|---|
| Grey literature search | The protocol primarily emphasized searches in major scientific databases. | In addition to the main databases, Google Scholar and the WHO Global Health Library were searched to capture grey literature. | This refinement was made to improve the comprehensiveness of the search and reduce publication bias by including non-indexed sources relevant to public health and disaster management. |
| Data synthesis approach | The protocol indicated that findings would be synthesized using narrative and thematic approaches. | A structured narrative synthesis with thematic categorization and visual evidence mapping was used, and an evidence-weighting framework was added to indicate the relative contribution of each study. | During analysis it became clear that included studies varied widely in design and outcome measures. The weighting framework was therefore introduced to improve transparency in interpreting heterogeneous evidence. |
| Study quality consideration | The protocol stated that study quality would be assessed using appropriate appraisal tools. | Two design-specific tools were used: ROBINS-I for non-randomized intervention studies and the JBI Critical Appraisal Checklist for observational studies. | The dual-tool approach allowed more appropriate methodological assessment according to study design. |
| Inclusion of simulation studies | The protocol allowed modelling studies relevant to real-world crowd management contexts. | Simulation and modelling studies were included if they evaluated preventive strategies applicable to real-world crowd safety scenarios. | This clarification ensured consistency with the review objective of identifying practical preventive strategies. |
| Evidence synthesis visualization | The protocol did not specify visualization tools. | The review included evidence mapping and graphical synthesis (Figures 2–5) to illustrate intervention domains, bias assessment, and study contribution. | These visualizations were added during analysis to improve clarity and interpretation of heterogeneous evidence. |
